# Influence of Prebiotic Fructans on Retronasal Aroma from Elderly Individuals

**DOI:** 10.3390/molecules26102906

**Published:** 2021-05-13

**Authors:** Carolina Muñoz-González, Marine Brule, Christophe Martin, Gilles Feron, Francis Canon

**Affiliations:** Centre for Taste and Feeding Behavior (CSGA), UMR1324 INRAe, UMR6265 CNRS University of Burgundy, Agrosup Dijon, F-21000 Dijon, France; marine.brule@live.fr (M.B.); christophe.martin@inrae.fr (C.M.); gilles.feron@inrae.fr (G.F.); francis.canon@inrae.fr (F.C.)

**Keywords:** prebiotic fibres, in vivo aroma release, PTR-MS, aroma perception, sweetness perception, dumping effect, personalized diets

## Abstract

This study investigates for the first time the role of fructans with prebiotic effects (oligofructose and inulin) on retronasal aroma among elderly individuals. The impact of oligofructose (20% *w*/*w*) on retronasal aroma release was investigated using proton transfer reaction-mass spectrometry (PTR-MS) after 73 elderly individuals consumed aqueous solutions aromatized with five aroma compounds (pentan-2-one, nonan-2-one, hexan-2,3-dione, octanal and linalool). The influence of oligofructose and inulin (10% *w*/*w*) on the perceived intensity (*n* = 26) of two aroma descriptors (butter and floral) was also studied together with the possibility of a dumping effect on aroma evaluation due to the sweetness provided by the fructans. The results showed that the presence of oligofructose produced a significant reduction in retronasal aroma release, which could be generally explained by the physicochemical properties of aroma compounds. The presence of prebiotic fructans did not significantly affect the perceived intensity of butter and floral notes, although a dumping effect for the butter descriptor in the presence of oligofructose was observed. To conclude, these findings suggest that although fructans can exert an impact on retronasal aroma, they can be used at precise concentrations to increase the prebiotic fibre content of food products without affecting the aroma profile of foods.

## 1. Introduction

Elderly individuals sometimes suffer from gastrointestinal problems, such as constipation, diverticulosis, upper gastrointestinal bleeding and colon cancers [1], that can negatively affect their quality of life. These pathologies might be promoted by nutritional changes [2], such as a reduced consumption of fibre [3] or non-starch carbohydrates, that result from changes in food habits and food preferences produced by disabilities related to oral processing of food or cognitive conditions. Thus, tooth loss, which is frequently experienced during ageing, may be the origin of difficulties in masticating specific food products such as vegetables, reducing appetence for these foods and thus dietary fibre intake [4]. Moreover, the reduced secretion of saliva (hyposalivation) produced by some medications or by changes in the functioning of salivary glands due to ageing [5] can affect mastication and food bolus formation [6], flavour release [7] and perception [8] and food consumption [8]. In this regard, a systematic literature review highlighted that salivary hypofunction is associated with a decrease in chewing and swallowing abilities and taste perception in elderly individuals [8]. Cognitive impairment in elderly individuals is associated with poor oral health [9] and reduced sensory perception [10], which can also affect food preferences and habits [8].

In this context, there is a need to develop strategies for personalized diets counterbalancing nutritional deficits while maintaining good sensory properties of foods. The utilization of functional fibres with prebiotic effects could be a powerful strategy to achieve this aim. Prebiotics are defined as substrates that are selectively utilized by host microorganisms, conferring a health benefit [11]. Some prebiotics normally used in the human diet are lactulose, galactooligosaccharides, fructooligosaccharides, inulin and its hydrolysates, among others [12]. Oligofructose and inulin are two of the most commonly used prebiotics [12] and belong to the fructan family. Inulin can be extracted from plants (e.g., chicory roots) and undergo partial hydrolysis to produce oligofructose. As they present a wide range of solubilities, they can be used to incorporate fibre in liquid systems such as drinks or dairy products [12], adding nutritional and health benefits such as the stimulation of probiotic bacteria [13,14,15,16,17,18]. Moreover, their addition to food products influences their sensory properties since they have a sweet character and allow the replacement of a considerable quantity of sugar [19] and/or fat [12], reducing the calorific content of products or increasing the stability of foams, among other technological advantages. 

The reformulation of food products with these ingredients not only influences food quality and taste but also might affect the rate and intensity of aroma release and perception (retronasal aroma) produced during food oral processing. Thus, when Siefarth and collaborators [20] evaluated the influence of oligofructose on aroma release in vitro, they found that the presence of this fructan retained most of the aroma compounds assayed within the solution, significantly limiting their release. However, in vivo, these effects can be modulated by food oral processing during food consumption, which is characteristic of each target population and can influence the final perception.

The present study aims to evaluate the effects of prebiotic fructans on the retronasal aroma of elderly individuals. In vivo release of five aroma compounds (pentan-2-one, nonan-2-one, hexan-2,3-dione, octanal and linalool) was followed by PTR-MS, while elderly individuals (*n* = 73) consumed an aromatized oligofructose solution (20% *w*/*w*) and a control aqueous solution. Moreover, elderly individuals (*n* = 26) rated the intensity perceived of two aroma descriptors (butter and floral) during the consumption of two fructan solutions (oligofructose and inulin at 10% *w*/*w*) with reference to a control aqueous solution. Additionally, the existence of a dumping effect on the evaluation of aroma intensity by the sweetness provided by the fructans was investigated.

## 2. Results and Discussion

### 2.1. Effects of Oligofructose on the In Vivo Aroma Release from Elderly Individuals

**Figure 1:**
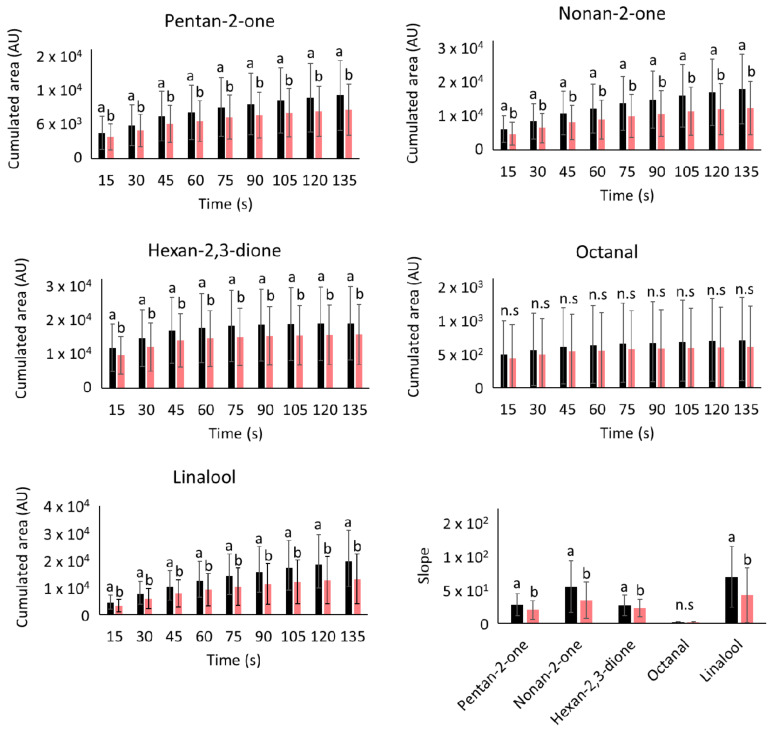
Effects of oligofructose on the retronasal release (cumulative area and slope) of five aroma compounds by 73 elderly individuals during consumption of a control aqueous solution (black) and an oligofructose solution (pink). Mean value ± standard deviation. Different letters (“a”, “b”) indicate significant differences among aqueous (control) and oligofructose solutions for each time slice of cumulated area (AU = arbitrary units) or for each compound regarding their slope (Tukey test, α < 0.05), and n.s., the difference was not significant.

The release of five aroma compounds (pentan-2-one, nonan-2-one, hexan-2,3-dione, octanal and linalool) resolved over time was followed in the noses of 73 elderly individuals during the consumption of aqueous solutions with the same aroma content but with different matrix compositions (control versus oligofructose solution). Figure 1 shows the cumulative area (CA) averaged over all of the individuals per 15-second time slice as well as the slope corresponding to the release of the last 20% of CA for the five aroma compounds that were assayed.

The addition of oligofructose (20% *w*/*w*) produced a lower retronasal release for all aroma compounds in comparison to the control solution (Figure 1). This indicates that a lower amount of aroma compounds reached the nasal cavity of the individuals after sample consumption in the presence of oligofructose compared to the control solution. This lower release was significant (*p* ≤ 0.05) for four (pentan-2-one, nonan-2-one, hexan-2,3-dione and linalool) of the compounds assayed but did not reach the significance level for octanal (*p* > 0.05). This lower release could influence aroma perception. To the authors’ knowledge, this is the first study that investigated the role of oligofructose in in vivo aroma release. However, Siefarth and coworkers [20] previously studied the effects of polyols and bulking agents, including oligofructose at 20% (*w*/*w*), on the in vitro release of four aroma compounds (cis-3-hexen-1-ol, benzaldehyde, ethyl butanoate and butyl isovalerate). For three of the four compounds assayed (benzaldehyde, ethyl butanoate and butyl isovalerate), the authors observed that the addition of oligofructose and the other carbohydrates tested decreased aroma release from aqueous solutions relative to pure water, which is in agreement with our findings. They proposed that nonvolatiles influence aroma release by providing a barrier to diffusion, resulting in volatiles being entrapped in the matrix. This effect could result from specific or nonspecific noncovalent interactions between nonvolatile and volatile compounds [21,22,23]. This explanation was also given by Hansson and coworkers [22] for the reduced aroma release observed in vitro in the presence of different carbohydrates (sucrose, invert sugar, and glucose syrup). 

Another factor that could explain the differential release in the presence of carbohydrates could be related to the viscosity of the solutions. However, Siefarth and collaborators [20], using similar experimental conditions, assayed here did not find a correlation between aroma release and viscosity, suggesting that the viscosity of the oligofructose solution at 20% (*w*/*w*) was not sufficiently high to exert an effect on aroma release. Indeed, it has been reported that aroma release decreases with viscosity only above the critical overlap concentration (C*), where there is a pronounced increase in viscosity [24]. However, below C*, aroma release seems to be independent of viscosity effects [24]. Nonetheless, it is important to consider that consumption is a dynamic process during which samples are subject to other phenomena, such as dilution with saliva, changes in temperature, and interaction with oral components such as the oral mucosa. Indeed, aroma compounds are prone to interact with the surface of the oral mucosa, influencing aroma persistence [25].

To investigate this hypothesis, the slope corresponding to the release of the last 20% of CA of the aroma compounds in both solutions, which represents the end of the kinetics, was measured (Figure 1). A higher slope indicates that aroma persistence was also higher. Four compounds (pentan-2-one, nonan-2-one, hexan-2,3-dione and linalool) presented a slope significantly different in the oligofructose solution compared to that of the control solution. Their slopes were significantly smaller in the oligofructose solution than in the control solution, indicating that the presence of the fibre decreased the persistence of aroma compounds in the mouth. This would indicate that in the presence of fibres, fewer aroma compounds have been adsorbed at the surface of the oral mucosa. Indeed, if the aroma compounds interact with the fibres, this interaction will shift the chemical equilibrium between free aroma compounds and bound aroma compounds at the surface of the mucosa, resulting in fewer aroma compounds bound at the mucosa surface. Thus, fewer aroma compounds will be desorbed from the mucosa surface over time. Nevertheless, new experiments monitoring longer times are necessary to confirm this point.

To better visualize the net retention effect exerted by oligofructose depending on the aroma compounds, the reduction in the quantity of aroma released due to the addition of oligofructose, expressed as a percentage, was calculated using the cumulative data corresponding to the 135 seconds of monitoring time after swallowing. These data together with the physicochemical properties of the aroma compounds are presented in Table 1. The reduction in the quantity of aroma released varied from 14% (octanal) to 33% (linalool) (Table 1). Thus, linalool, the most hydrophobic compound with a higher molecular weight, showed the lowest release, while the compound octanal, also very hydrophobic and with a high molecular weight, was less affected by the presence of oligofructose compared to the control solution (Table 1). The lower effect observed for octanal is in agreement with the fact that this compound was the only one not significantly affected by the presence of oligofructose. Considering the three other compounds that all belong to the ketone family, we observed a correlation between the hydrophobicity (log P) of the compounds and the amplitude of decrease in the release: hexan-2,3-dione (release −17%/log P −0.35), pentan-2-one (release −23%/log P 0.75) and nonan-2-one (release −31%/log P 2.70). Considering linalool and the three ketones, we obtained a linear correlation between the log P values and the decrease in release with the following parameters: r = 0.998 and r^2^ = 0.995. With the exception of octanal, these results are in agreement with the findings previously observed in vitro by other authors, who found a relationship between the physicochemical properties of aroma compounds (and mostly molecular weight, hydrophobicity and volatility) and their retention in the presence of carbohydrates [20,23,26,27,28]. In contrast to in vitro experiments, this experiment was performed under in vivo conditions, and thus other factors related to the oral transfer of the solutions could have affected the behaviour of octanal, such as its metabolization in the mouth [25,29].

### 2.2. Effects of Prebiotic Fructans (Oligofructose and Inulin) on Aroma Perception in Elderly Individuals

**Figure 2:**
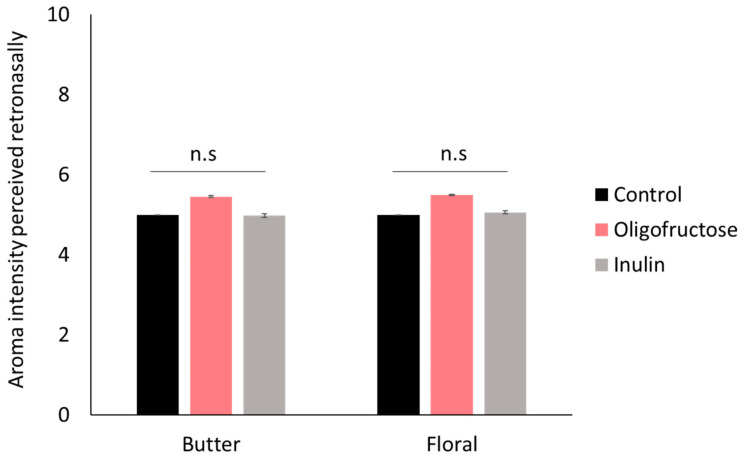
Retronasal perception of butter and floral notes perceived by 26 elderly individuals in solutions with prebiotic fibres added (oligofructose, inulin) in comparison to a control sample with no matrix added. Mean value ± standard error (*p* > 0.05).

Hexan-2,3-dione and linalool were retained for sensory experiments, as both seem to react as a function of their physicochemical properties and represent two extreme values. Hexan-2,3-dione and linalool have butter and floral odours, respectively. The effects of fructans (oligofructose and inulin) on the intensity of butter and floral descriptors perceived by 26 elderly individuals were evaluated by one-way ANOVA (Figure 2). The evaluation of the two aroma descriptors was performed independently of each other. Figure 2 shows that there were no significant differences between the control and the samples with fibres added (oligofructose and inulin) for the perception of butter and floral notes. Additionally, there was no effect of the degree of polymerization (DP) of fructans on aroma perception (Figure 2).

Indeed, no significant differences in aroma perception between inulin (with a higher DP) and oligofructose were observed, although the latter presented slightly higher intensity aroma scores than the former for both attributes. Therefore, although it has been reported that the presence of matrix ingredients with larger molecular weights and volumes in solution would produce solutions with higher viscosities [30], which could affect aroma release, the differences in DP between oligofructose and inulin at the concentrations assayed here were not important enough to significantly affect the perception of aroma compounds. This result suggests that prebiotic fructans at this concentration could be used to reinforce the fibre content of foods without changing their aroma profile.

**Figure 3:**
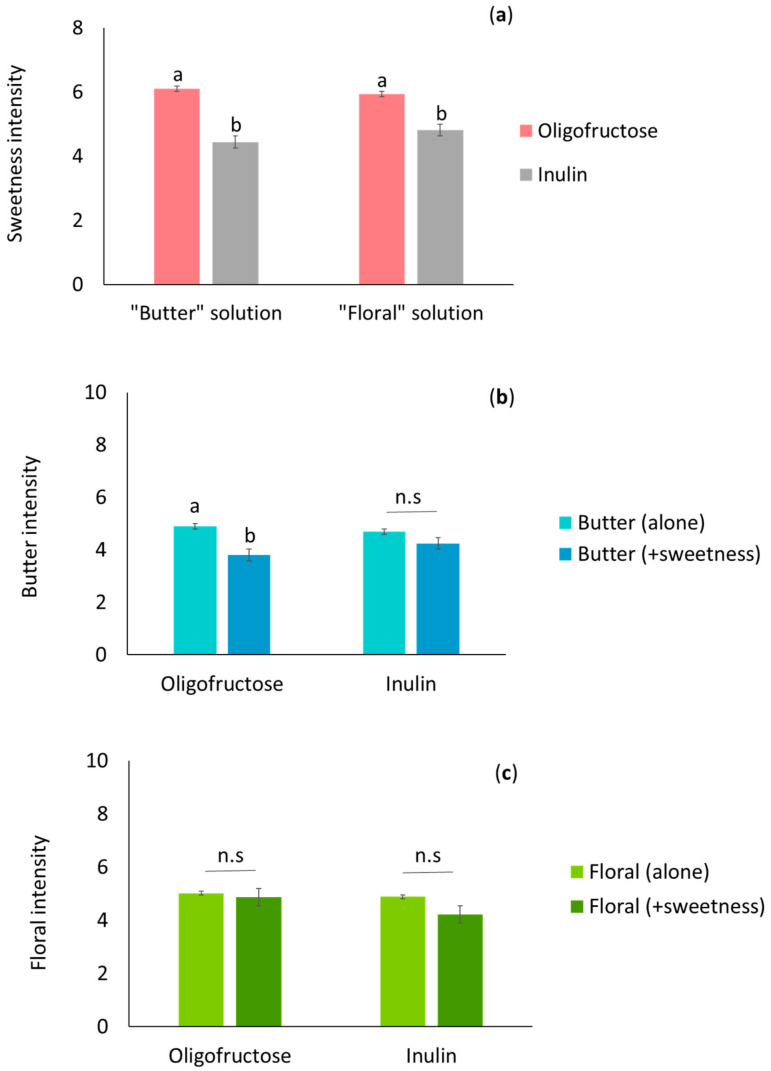
Intensity scores of the sweetness (**a**), butter (**b**) and floral (**c**) descriptors determined retronasally by 26 elderly individuals in the oligofructose and inulin solutions aromatized with butter or floral descriptors (hexan-2,3-dione and linalool, respectively). Mean value ± standard error. Different letters (“a”, “b”) indicate significant differences for a taste or aroma descriptor between solutions (Tukey test, α < 0.05), and n.s. indicates the difference was not significant.

This result differs from the lower retronasal release observed in the instrumental experiments for the oligofructose solutions. Although comparison between both experiments must be performed with caution since the oligofructose concentration was different in both experiments (20% versus 10% *w*/*w*), a divergence between retronasal release and aroma perception of beverages in the presence of sugar has been previously observed [31]. The authors stated that sensory interactions between aroma and sweet perception could be the reason for these divergences. Indeed, flavour perception is a multimodal perception. Thus, taste and aroma perception can interact at a perceptual level to contribute to the overall sensory property of the solution. As a result, the perception of a sweet taste can reinforce the perception of aroma. Since the prebiotic fibres used in this study have a sweet character, we further evaluated the potential existence of a dumping effect of sweetness on aroma perception. Individuals were asked to rate the aroma intensity of the samples with prebiotic fibres added under two conditions: evaluation of aroma intensity of the samples (aroma alone) or evaluation of aroma and sweetness intensity of the samples at the same time (aroma + sweetness) (Figure 3). Figure 3a shows the sweetness intensity scores given by the individuals to the solutions. The oligofructose solution presented a significantly more intense sweetness level than the inulin solution regardless of the aroma descriptor assayed. This finding is in agreement with the chemical structure of the fructans, since a large degree of polymerization of fructans is associated with a lower sweetness perception, with oligofructose having a sweeter character than inulin.

The intensity of butter was rated more intensely (*p* = 0.50) by individuals when they were asked to rate only the aroma (butter) intensity of the solution than when they were asked to rate both the butter and sweetness intensity of the oligofructose solution. This means that individuals tended to overcompensate for the intensity of this aroma descriptor when they evaluated it without considering the other predominant flavour attribute of the solution (sweetness). This result is in line with the findings of Clark and Lawless [32], who found a dumping effect between sweetness and flavour intensity in beverages. However, this effect was not found in the inulin solution, probably because the sweetness level contributed by inulin was lower than that of oligofructose. The floral intensity (Figure 3c was not affected by a dumping effect in either the oligofructose or the inulin solution (Figure 3). The dumping effect observed for the butter note could also partially explain the difference observed between the analytical and sensory results.

## 3. Materials and Methods

The French Ethics Committee for Research approved this study (CPP Est I. Dijon, #14.06.03, ANSM #2014-A00071-46). Individuals received oral and written information on the study and gave written informed consent before participating. They received financial compensation for their participation.

### 3.1. Ingredients

For this study, two carbohydrates with known prebiotic effects were used to evaluate their influence on five relevant food-grade aroma compounds (pentan-2-one, nonan-2-one, hexan-2,3-dione, octanal and linalool) (Sigma-Aldrich, Saint Quentin Fallavier, France). They belong to the family of fructans but present different degrees of polymerization. Inulin (Orafti^®^ GR) presents an average degree of polymerization (DP) ≥ 10 (92% purity), while oligofructose (Orafti^®^ P95) is obtained by hydrolysis of inulin, and its DP is 2–8 (95% purity). Both fructans were kindly donated by Beneo (Tienen, Belgium).

### 3.2. In Vivo Retronasal Aroma Release by PTR-ToF-MS

Seventy-three elderly individuals living independently in Dijon (40 women and 33 men, age: 73.99 ± 5.46 years old, body mass index (BMI): 28.03 ± 4.85 kg/m^2^) were selected from the AlimaSSens project (https://anr.fr/Projet-ANR-14-CE20-0003, accessed on 12 May 2021) based on their good physical and mental status. Individuals were not allowed to smoke, eat or drink at least one hour before the sessions.

Individuals were invited to attend the Centre for Taste and Feeding Behaviour (Dijon, France), where they consumed two aqueous solutions following specific instructions. The solutions were an aromatized aqueous solution without (control) or with 20% (*w*/*w*) oligofructose (oligofructose). The aromatized solutions contained a mixture of the five aroma compounds at the following concentrations: pentan-2-one (1 ppm), nonan-2-one (5 ppm), hexan-2,3-dione (20 ppm), octanal (3 ppm) and linalool (40 ppm). The samples were prepared immediately prior to the in vivo analyses. Individuals were instructed to introduce the entire sample (10 mL) into their mouths and keep it for 30 seconds before swallowing it. After that, individuals were instructed to swallow their saliva every 30 s for 2.5 min. The samples were evaluated on two different days (once per day). The rate and amount of the compounds that passed from their mouth to their nasal cavities were measured by means of PTR-ToF-MS (PTR-ToF 8000, Ionicon Analytik, Innsbruck, Austria), as described previously [33]. All breath data acquired were analysed using IGOR Pro (WaveMetrics, Inc. Portland, USA).

### 3.3. Sensory Analyses

Twenty-six elderly individuals living independently in Dijon (13 women and 13 men, age: 72.73 ± 4.97 years old, BMI: 27.56 ± 4.67 kg/m^2^) were selected from the AlimaSSens project (https://anr.fr/Projet-ANR-14-CE20-0003, accessed on 12 May 2021) based on their good physical and mental status. All individuals had a normal sense of smell on the basis of the European test of olfactory capabilities (panel score = 14.6 out of 16), which measures the olfactory capability of individuals for the detection of 16 odours [34]. Individuals were not allowed to smoke, eat or drink starting at least one hour before the different test sessions.

Sensory sessions took place in an air-conditioned (21 ± 2 °C) sensory testing room of the ChemoSens platform (Centre for Taste and Feeding Behaviour, Dijon, France) using standardized booths equipped with computers. All sessions were performed using Fizz^®^ software. For the sensory analyses, two odourants (hexan-2,3-dione and linalool) were independently evaluated. The solutions were prepared immediately prior to the sensory sessions by diluting the stock solutions in water (Evian, France). The procedure of consumption was as follows: individuals were asked to introduce the sample into their mouths with the nose closed, keep it in the mouth for 5 seconds, unclose the nose and swallow the entire sample, and then evaluate the sample.

Individuals were trained in the recognition of the descriptors “butter” for hexan-2,3-dione and “floral” for linalool. It was also determined if individuals were able to discriminate the retronasal aroma intensity in a control solution with different concentrations of aroma compounds added (1, 3, 9, 27 ppm) by means of intensity ranking tests and unstructured intensity scales delimited at the ends (0 to 10 cm; 0 = not very intense, 10 = very intense). For the evaluation session, the compounds linalool and hexan-2,3-dione were diluted at a concentration of 9 ppm in three aqueous solutions consisting of an aromatized aqueous solution (i) without (control), (ii) with 10% (*w*/*w*) oligofructose added (oligofructose) or (iii) 10% (*w*/*w*) inulin added (inulin). Individuals were asked to evaluate the aroma intensity of the samples with fructans added with respect to that of a reference. The reference corresponded to the control sample whose intensity was arbitrarily ranked in the middle of the evaluation scale, corresponding to an intensity of 5. Samples were measured in duplicate. To evaluate the presence of a potential dumping effect [35] of sweetness, which is the predominant taste descriptor provided by fructans, on aroma evaluation, individuals were asked to evaluate the aroma intensity of the samples with prebiotic fructans added in two conditions. In the first condition, individuals were asked to evaluate only the aroma intensity of the samples using one intensity scale (aroma alone). In the second condition, individuals were asked to evaluate both the aroma intensity and the intensity of sweetness perceived using two intensity scales (aroma + sweetness). Individuals were not informed about the aim of this experiment.

### 3.4. Statistical Analyses

One-way analysis of variance (ANOVA) and Tukey’s test for mean comparison were used to assess the influence of fructan addition on in vivo aroma release and perception and to evaluate the presence of the dumping effect. Linear regression was performed to establish the relationship between log *p* values of aroma compounds and the percentage of retention on in vivo aroma release due to the presence of oligofructose. The significance level was *p* ≤ 0.05 throughout the study. The XLSTAT program (v.19.01) (Addinsoft, Paris, France) was used for data processing.

## 4. Conclusions

This study aimed to evaluate for the first time the effects of fructans with prebiotic effects on retronasal aroma in a panel of elderly individuals. Oligofructose (20% *w*/*w*) reduced the in vivo release of the five aroma compounds assayed (pentan-2-one, nonan-2-one, hexan-2,3-dione, octanal and linalool), and this reduction was compound-dependent and ranged from 14 to 33% for octanal and linalool, respectively. The effects of oligofructose on the in vivo release of aroma compounds could be partially explained by their physicochemical properties, since in general, aroma compounds with higher hydrophobicity values had lower in vivo release in the presence of oligofructose compared to the control solution. This suggests that hydrophobic effects may drive the interaction between oligofructose and aroma compounds. However, octanal did not follow this correlation and was the only compound assayed in which the reduction in the in vivo release by the presence of oligofructose did not reach the significance level. Oligofructose and inulin (10% *w*/*w*) did not affect significantly the retronasal aroma intensity of butter and floral descriptors (elicited by hexan-2,3-dione and linalool, respectively), which would indicate that they can be added to food products without changing their aroma profile. However, a dumping effect was observed for the butter note intensity in the presence of oligofructose, with lower ratings of butter intensity when evaluated together with sweetness intensity than when evaluated alone. The results from this study suggest that fructans can exert an effect on retronasal aroma depending on the aroma compound structure, type of fibres and concentrations. These results also suggest that under certain conditions, it is possible to fortify foods with fructans without changing aroma perception. These pioneering results necessitate further studies for confirmation.

## Figures and Tables

**Table 1:** Physicochemical characteristics of the aroma compounds assayed and reduction in the quantity of aroma released due to the addition of oligofructose, expressed as a percentage.

Aroma Compounds	MW ^a^ (g/mol)	Log P ^b^	BP ^c^ (°C)	Reduction in Aroma Released (%)
**Hexan-2,3-dione**	114	−0.35	162	17.29 ab
**Pentan-2-one**	86	0.75	95	23.02 ab
**Nonan-2-one**	142	2.70	185	31.22 ab
**Octanal**	128	2.80	176	13.56 b
**Linalool**	154	2.97	204	32.55 a

^a^ Molecular weight (g/mol). ^b^ Hydrophobic constant estimated with the EPI suite (US EPA 2000–2007). ^c^ Boiling point (°C) estimated with EPI Suite (US EPA 2000–2007). Different letters (“a”, “b”) denote significant differences on the in vivo release of aroma compounds in the oligofructose solution with respect to the aqueous (control) solution (Tukey’s test, α < 0.05).

## Data Availability

The data presented in this study are available upon request from the corresponding author.

## Sample Availability

Samples of the compounds are available from the authors.

## References

[1] Tiihonen K., Ouwehand A.C., Rautonen N. (2010). Human intestinal microbiota and healthy ageing. Ageing Res. Rev..

[2] Comparato G., Pilotto A., Franzè A., Franceschi M., Di Mario F. (2007). Diverticular disease in the elderly. Dig. Dis..

[3] Laurin D., Brodeur J.M., Bourdages J., Vallee R., Lachapelle D. (1994). Fibre intake in elderly individuals with poor masticatory performance. J. Ca. Dent. Assoc..

[4] Yoshida M., Kikutani T., Yoshikawa M., Tsuga K., Kimura M., Akagawa Y. (2011). Correlation between dental and nutritional status in community-dwelling elderly Japanese. Geriatr. Gerontol. Int..

[5] Affoo R.H., Foley N., Garrick R., Siqueira W.L., Martin R.E. (2015). Meta-analysis of salivary flow rates in young and older adults. J. Am. Geriatr. Soc..

[6] Peyron M.A., Santé-Lhoutellier V., François O., Hennequin M. (2018). Oral declines and mastication deficiencies cause alteration of food bolus properties. Food Funct..

[7] Muñoz-González C., Brulé M., Feron G., Canon F. (2019). Does interindividual variability of saliva affect the release and metabolization of aroma compounds ex vivo? The particular case of elderly suffering or not from hyposalivation. J. Texture Stud..

[8] Muñoz-González C., Vandenberghe-Descamps M., Feron G., Canon F., Labouré H., Sulmont-Rossé C. (2018). Association between Salivary Hypofunction and Food Consumption in the Elderlies. A Systematic Literature Review. J. Nutr. Health Aging.

[9] Kossioni A.E. (2018). The association of poor oral health parameters with malnutrition in older adults: A review considering the potential implications for cognitive impairment. Nutrients.

[10] Aliani M., Udenigwe C.C., Girgih A.T., Pownall T.L., Bugera J.L., Eskin M.N.A. (2013). Aroma and Taste Perceptions With Alzheimer Disease and Stroke. Crit. Rev. Food Sci. Nutr..

[11] Gibson G.R., Hutkins R., Sanders M.E., Prescott S.L., Reimer R.A., Salminen S.J., Scott K., Stanton C., Swanson K.S., Cani P.D. (2017). Expert consensus document: The International Scientific Association for Probiotics and Prebiotics (ISAPP) consensus statement on the definition and scope of prebiotics. Nat. Rev. Gastroenterol. Hepatol..

[12] Al-Sheraji S.H., Ismail A., Manap M.Y., Mustafa S., Yusof R.M., Hassan F.A. (2013). Prebiotics as functional foods: A review. J. Funct. Foods.

[13] Tayyebymoghadam S E.M. (2020). Comparison of the effect of extracted inulin from native chicory root with commercial inulin on the viability of probiotics and physicochemical, rheological and sensory properties of synbiotic yogurt. JFST.

[14] Majuwana K., Menaka G., Kariyawasam M., Lee N., Paik H. (2021). Food Bioscience Synbiotic yoghurt supplemented with novel probiotic Lactobacillus brevis KU200019 and fructooligosaccharides. Food Biosci..

[15] Dolores M., Iriondo-dehond A., Iriondo-dehond M., Gonzalez I., Medrano A., Filip R., Uribarri J. (2020). Healthy eating recommendations: Good for reducing dietary contribution to the body’s advanced glycation/lipoxidation end products pool?. Nutr. Res. Rev..

[16] Chand P., Kumar M.D., Singh A.K., Deshwal G.K., Rao P.S., Tomar S.K., Sharma H. (2021). Low-calorie synbiotic yoghurt from indigenous probiotic culture and combination of inulin and oligofructose: Improved sensory, rheological, and textural attributes. J. Food Process. Preserv..

[17] Al-shawi S.G., Dang D.S., Yousif A.Y., Al-younis Z.K., Najm T.A., Matarneh S.K. (2020). The Potential Use of Probiotics to Improve Animal Health, Efficiency, and Meat Quality: A Review. Agriculture.

[18] Yousefvand M.G.A., Zarei M., Farhangnia P. (2020). Developing novel synbiotic low-fat yogurt with fucoxylogalacturonan from tragacanth gum: Investigation of quality parameters and Lactobacillus casei survival. Food Sci. Nutr..

[19] Ruiz-Aceituno L., Hernandez-Hernandez O., Moreno F.J., Methven L. (2018). Sweetness and sensory properties of commercial and novel oligosaccharides of prebiotic potential. LWT Food Sci. Technol..

[20] Siefarth C., Tyapkova O., Beauchamp J., Schweiggert U., Buettner A., Bader S. (2011). Influence of polyols and bulking agents on flavour release from low-viscosity solutions. Food Chem..

[21] Carr J., Baloga D., Guinard J., Lawter L., Marty C., Squire C. (1996). The Effect of Gelling Agent Type and Concentration on Flavor Release in Model Systems. Flavor-Food Interactions.

[22] Hansson A., Andersson J., Leufve A. (2001). The efect of sugars and pectin on flavour release from a soft drink-related model system. Food Chem..

[23] Hilippe E.L.P., Euvre A.N., Olas B.E.C., Angendorff V.I.L., Chippa C.H.S., Ndre A., Oilley Ä.E.V. (2003). Behavior of Flavor Compounds in Model Food Systems: A Thermodynamic Study. J. Agric. Food Chem..

[24] Malone M.E., Appelqvist I.A.M., Norton I.T. (2003). Oral behaviour of food hydrocolloids and emulsions. Part 2. Taste and aroma release. Food Hydrocoll..

[25] Ployon S., Brulé M., Andriot I., Morzel M., Canon F. (2020). Understanding retention and metabolization of aroma compounds using an in vitro model of oral mucosa. Food Chem..

[26] Pangborn R.M., Szczesniak A. (1974). Effect of hydrocolloids and viscosity on flavor and odor intensities of aromatic flavor compounds. J. Texture Stud..

[27] Nahon D.F., Roozen J.P., Posthumus M.A. (1998). Flavor Release from Mixtures of Sodium Cyclamate, Sucrose, and an Orange Aroma. J. Agric. Food Chem..

[28] Goubet I., Voilley A.J. (1998). Retention of Aroma Compounds by Carbohydrates: Influence of Their Physicochemical Characteristics and of Their Physical State. J. Agric. Food Chem..

[29] Muñoz-gonzález C., Feron G., Brulé M., Canon F. (2018). Understanding the release and metabolism of aroma compounds using micro-volume saliva samples by ex vivo approaches. Food Chem..

[30] Roberts D.D., Acree T.E. (1996). Effects of Heating and Cream Addition on Fresh Raspberry Aroma Using a Retronasal Aroma Simulator and Gas Chromatography Olfactometry. J. Agric. Food Chem..

[31] Saint-Eve A., Déléris I., Aubin E., Semon E., Feron G., Rabillier J.M., Ibarra D., Guichard E., Souchon I. (2009). Influence of composition (CO_2_ and sugar) on aroma release and perception of mint-flavored carbonated beverages. J. Agric. Food Chem..

[32] Clark C.C., Lawless H.T. (1994). Limiting response alternatives in time-intensity scaling: An examination of the halo-dumping effect. Chem. Senses.

[33] Muñoz-González C., Feron G., Canon F. (2021). Physiological and oral parameters contribute prediction of retronasal aroma release in an elderly cohort. Food Chem..

[34] Thomas-Danguin T., Rouby C., Sicard G., Vigouroux M., Farget V., Johanson A., Bengtzon A., Hall G. (2003). Development of the ETOC: A European Test of Olfactory Capabilities. Rhinology.

[35] Abdi H. (2002). What can cognitive psychology and sensory evaluation learn from each other?. Food Qual. Prefer..

